# Zuschreibungsprozesse des vierten Alters: Abgrenzungs- und Aufwertungsstrategien im Umgang mit sozialer Abwertung

**DOI:** 10.1007/s00391-026-02610-0

**Published:** 2026-07-13

**Authors:** Rebekka Steinlechner

**Affiliations:** Kompetenzzentrum für Gerontologie und Gesundheitsforschung, Karl Landsteiner Privatuniversität, Dr.-Karl-Dorrek-Straße 30, 3500 Krems an der Donau, Österreich

**Keywords:** Soziale Kategorisierung, Soziale Identität, Relationale Autonomie, Selbstbild, Informationsmanagement, Social categorization, Social identity, Relational autonomy, Self-concept, Information management

## Abstract

**Hintergrund:**

Das vierte Alter bezeichnet eine Lebensphase, die durch Abhängigkeit, Pflegebedürftigkeit und den Verlust der Identität gekennzeichnet ist. Durch die negative Konnotation dieser Lebensphase wird eine Zugehörigkeit von den Betroffenen abgelehnt, weshalb Gilleard und Higgs das vierte Alter als soziales Imaginär bezeichnen, das lediglich durch die Fremdzuschreibung realisiert wird.

**Ziel:**

Der Beitrag verfolgt die Fragestellungen, wie Menschen im vierten Alter mit der sozialen Abwertung umgehen und ein positives Selbstbild herstellen.

**Methoden:**

Es wurden 16 problemzentrierte Interviews im Zeitraum von 2019 bis 2020 mit älteren Österreicher:innen (78 bis 98 Jahre), die mehrere gesundheitliche Einschränkungen aufweisen, geführt und mit der dokumentarischen Methode ausgewertet.

**Ergebnisse:**

Ältere Menschen mit mehreren Einschränkungen versuchen, sich von der stark negativ konnotierten Gruppe des vierten Alters abzugrenzen und für sich selbst eine positive soziale Identität aufrechtzuerhalten. Dazu konnten vier Strategien festgestellt werden: (1) der Vergleich mit anderen älteren Menschen, um die eigene Situation aufzuwerten, (2) die Neuinterpretation von Autonomie in Relation zur Unterstützung durch Andere, (3) die Betonung der eigenen Leistungsfähigkeit und (4) die selektive Kommunikation des eigenen Befindens.

**Diskussion:**

Diese Strategien ermöglichen es den Befragten, weiterhin Teil einer positiv bewerteten Gruppe von abhängigen, aber autonomen Älteren zu bleiben. Das vierte Alter lässt sich daher auch bei Menschen im vierten Alter als soziales Imaginär ausmachen, das anderen zugeschrieben wird.

## Abstract (en)

**Background:**

The fourth age refers to a stage of life characterized by dependency, need for care and loss of identity. Due to the negative connotations associated with this stage of life, those affected reject identifying with it. Therefore, Gilleard and Higgs describe it as a social imaginary that is realized only through attribution by others.

**Objective:**

This paper addresses the following questions: how do people in the fourth age deal with social devaluation and maintain a positive self-image?

**Methods:**

In this stud 16 problem-centered interviews were conducted between 2019 and 2020 with older Austrians (aged 78–98 years) who had multiple health impairments and were analyzed using the documentary method.

**Results:**

Older people with multiple impairments try to distance themselves from the negatively connoted group of care-dependent people in the fourth age and to maintain a positive social identity for themselves. In this context four strategies were identified: (1) comparing themselves with other older people in order to enhance their own situation, (2) reinterpreting autonomy in relation to support from others, (3) emphasizing their own capabilities and (4) selectively communicating their own well-being.

**Conclusion:**

These strategies enable respondents to remain part of a positively valued group of dependent yet autonomous older people. The fourth age can therefore also be identified among people in the fourth age as a social imaginary.

Die Lebensphase des vierten Alters wird als Bedrohung für die eigene Identität und Autonomie erlebt und daher eine Zugehörigkeit abgelehnt. Im Sinne der Theorie von Tajfel entsteht ein positives Selbstbild von Menschen im vierten Alter durch Vergleichs- und Aufwertungsprozesse, die eine Abgrenzung der eigenen Gruppe zur Gruppe der pflegebedürftigen Anderen ermöglichen. Daher kommen insbesondere der Darstellung und dem Erleben von Autonomie und Selbstständigkeit große Bedeutung zu.

## Hintergrund

In der Sozialgerontologie wird das Alter in mindestens zwei Lebensphasen unterteilt [19, 24]. Relativ unabhängig vom kalendarischen Alter zeichnet sich dabei das dritte, junge Alter durch Autonomie, Gesundheit und soziale Teilhabe aus [11], während das vierte Alter durch Fragilität, Vulnerabilität und den Verlust der Identität gekennzeichnet ist [9]. Das vierte Alter wird als Negativfolie zum dritten Alter konstruiert und umfasst dementsprechend alle älteren Menschen, die nicht aktiv und erfolgreich altern konnten [10, 24]. Insbesondere aufgrund von körperlichen und/oder geistigen Einschränkungen werden ältere Menschen von anderen Personen, wie etwa von Angehörigen oder Gesundheitspersonal, dem vierten Alter zugeordnet und erfahren damit eine soziale Abwertung [9]. Viele Studien zeigen daher, dass ältere Menschen mit mehreren Einschränkungen eine Zugehörigkeit zum vierten Alter ablehnen [1, 3, 15, 25].

Bisherige Studien haben sich damit beschäftigt, wie Menschen im vierten Alter ihre Identität trotz dieser defizitären Diskurse aufrechterhalten. Einerseits versuchen ältere Menschen, die Kontinuität ihres Selbstbildes zu bewahren, indem sie z. B. alltäglichen Aufgaben und sozialen Aktivitäten auch in abgeändertem Maße weiterhin nachgehen [15, 25] oder sich auf gewisse Tätigkeiten fokussieren, die sie ohne Unterstützung ausüben können [14].

Andererseits können sie sich auch über eine Neubewertung ihrer Situation ein angepasstes Selbstbild schaffen. Dies geschieht etwa über den Vergleich mit anderen älteren Menschen, denen es schlechter geht [16, 25] oder über eine neues Verständnis von Autonomie [12]. Lloyd et al. [12] betonen dabei die Bedeutung von relationaler Autonomie: Das bewusste Annehmen und auch das Ablehnen von Unterstützung durch andere Menschen wird zum Ausdruck von autonomen Handeln [12, 13, 18].

Ein weiterer Forschungsstrang beschäftigt sich mit dem sozialen und emotionalen Erleben von Fragilität im hohen Alter. Aufgrund der negativen Vorstellungen von Gebrechlichkeit versuchen ältere Menschen, sich nicht als fragil und damit vulnerabel zu verstehen, und beschreiben Fragilität eher als eine temporäre Erfahrung, also als einen gewissen Zeitpunkt, zu dem sie sich gebrechlich fühlen [6, 7]. Ebenfalls wird das Gefühl der Gebrechlichkeit auf den Körper reduziert und damit die Identität vom Körper getrennt wahrgenommen, um das Selbstbild trotz körperlicher Einschränkungen aufrechtzuerhalten [20].

Um ein besseres Verständnis davon zu erhalten, wie ältere Menschen mit Einschränkungen eine soziale Abwertung durch die wahrgenommene Zugehörigkeit zum vierten Alter verhindern, werden im Folgenden – basierend auf der Theorie der sozialen Identität von Tajfel [22] – die Abgrenzungs- und Aufwertungsstrategien näher analysiert. Der Beitrag verfolgt die Fragestellungen, wie Menschen im vierten Alter mit der sozialen Abwertung umgehen und für sich ein positives Selbstbild herstellen.

## Soziale Kategorisierung und Identität im vierten Alter

Gilleard und Higgs haben sich in ihren Arbeiten mit dem vierten Alter auseinandergesetzt und beschreiben es weniger als eine eigene Altersgruppe oder Lebensphase und mehr als „a state of ‚unbecoming‘“ [8, S. 13]. Sie definieren das vierte Alter als soziales Imaginär, das von außen – also von Menschen, die sich nicht im vierten Alter befinden – imaginiert wird, und dem all das zugeschrieben wird, was von der westlichen Leistungsgesellschaft abgelehnt wird, nämlich Abhängigkeit, Leistungseinbußen und Pflegebedürftigkeit [9, 10]. Das vierte Alter existiert daher als „alternative imaginary of old age, realised through the objectifying narratives and practices of others“ [8, S. 14]. Der Eintritt in das vierte Alter geschehe nun durch die Fremdzuschreibung von Fragilität und von Vulnerabilität gegenüber weiteren Einschränkungen und Verlusten. Der ältere Mensch wird dabei ohne Handlungsmacht und Subjektstatus imaginiert mit kaum einer Möglichkeit, sich dieser sozial abgewerteten Position zu widersetzten [8, 9].

Um analysieren zu können, wie Menschen, die dem vierten Alter von Anderen zugeordnet werden, mit dieser Kategorisierung umgehen, wird die Theorie der sozialen Identität von Tajfel [22] herangezogen. Diese erklärt, dass ein Teil der persönlichen Identität aus ihrer Zugehörigkeit zu sozialen Gruppen entsteht. Menschen neigen dazu, ihre soziale Umwelt zu ordnen, indem sie sich selbst und andere basierend auf sozialen Kategorien, wie Alter, Geschlecht oder Herkunft, in Gruppen einteilen bzw. auch von anderen eingeteilt werden.

Wenn sich die Individuen nun mit der Gruppe identifizieren, der sie zugeordnet werden, wird die Gruppenzugehörigkeit Teil des Selbstkonzeptes, und sie übernehmen Werte, Einstellungen, Verhalten und das Selbstbild. Eine Gruppenzugehörigkeit wird nur dann angenommen, wenn sie mit einer positiv besetzten, sozialen Identität einhergeht. Diese Positivität entsteht durch soziale Vergleiche, indem die eigene Gruppe aufgewertet und die Vergleichsgruppe abgewertet wird.

Es kann jedoch vorkommen, dass die zugeordnete Gruppe im Vergleich zu anderen Gruppen negativ wahrgenommen wird und eine Identifikation abgelehnt wird. In diesem Fall kann versucht werden, die Gruppe zu verlassen oder die Attribute der Gruppe neu und aufwertend zu interpretieren. Ebenfalls kann versucht werden, den Status der Gruppe aktiv zu verbessern. Alle diese Strategien dienen dazu, den eigenen Selbstwert trotz negativer Gruppenzuschreibungen zu schützen oder zu erhöhen [22, 23].

## Methodik

Zwischen Juli 2019 und Mai 2020 wurden in Österreich insgesamt 16 problemzentrierte Interviews nach Witzel [26] durchgeführt. Da in der Theorie der Eintritt ins vierte Alter durch Fremdzuschreibungen geschieht, wurden die Teilnehmenden auf Basis der Erzählungen der Gatekeeper ausgewählt. Das zentrale Kriterium für die Auswahl waren Erzählungen von mehreren gesundheitlichen Einschränkungen, wobei zwei Personen mit subjektiv guter Gesundheit zur Kontrastierung interviewt wurden. Das einzige Ausschlusskriterium waren schwere kognitive Beeinträchtigungen. Die Rekrutierung erfolgte über frühere Projekte, Seniorenclubs, kirchliche Verbände, Gemeinden sowie über Schneeballverfahren.

Alle Interviews wurden vor dem Ausbruch der COVID-19-Pandemie persönlich durchgeführt – mit Ausnahme des letzten Interviews im Mai 2020, das aufgrund der Situation telefonisch stattfinden musste. Ein Interview wurde als Paarinterview geführt. Die Studie wurde mit der Zustimmung der zuständigen Ethikkommission durchgeführt, und alle beteiligten Personen erteilten ihre schriftliche Einwilligung.

Im Durchschnitt dauerten die Interviews 94 min. Der Leitfaden des problemzentrierten Interviews begann mit der Bitte, den Ablauf des gestrigen Tages zu schildern. Als allgemeine Vertiefungsstrategie wurden Fragen zu weiteren Alltagsaktivitäten, Veränderungen im Alltag, Gesundheit und Wohnsituation und Zukunftsplänen gestellt.

Die Interviewten sind zwischen 78 und 98 Jahre alt, mit einem Durchschnittsalter von 84 Jahren. Elf der Befragten sind Frauen, fünf sind Männer (Tab. 1). Sieben Interviewte haben einen niedrigen Bildungsabschluss, und fünf verfügen über eine monatliche Pension von weniger als 1000 €. Neun Befragte leben allein, eine Person lebt in einem Mehrgenerationenhaushalt mit Kindern und Enkelkindern. Die übrigen leben mit ihrem Partner bzw. ihrer Partnerin zusammen. Eine Person ist kinderlos, die anderen haben zwischen ein und vier Kindern. Die Interviewten wurden aus fünf verschiedenen Bundesländern sowie aus ländlichen, halbländlichen und urbanen Regionen rekrutiert.

**Tab. 1:** Beschreibung der Interviewpartner:innen

Fall	Alter (Jahre)	Geschlecht	Funktionale Einschränkungen
IP1 Heidi	75–79	Weiblich	Moderat
IP2 Elsbeth	80–84	Weiblich	Leicht
IP3 Brunhild	80–84	Weiblich	Moderat
IP4 Gudrun	75–79	Weiblich	Schwer
IP5 Gertrud	80–84	Weiblich	Moderat
IP6 Karl	85–89	Männlich	Schwer
IP7 Alma	80–84	Weiblich	Schwer
IP8 Theresa	85–89	Weiblich	Schwer
IP9 Stefanie	80–84	Weiblich	Leicht
IP10 Hilde	85–89	Weiblich	Schwer
IP11 Gerald	85–89	Männlich	Moderat
IP12 Hans	80–84	Männlich	Moderat
IP13 Franz	80–84	Männlich	Schwer
IP14 Maria	80–84	Weiblich	Schwer
IP15 Herbert	90–94	Männlich	Moderat
IP16 Selina	95–99	Weiblich	Moderat

Die hier dargestellte Einschätzung der Einschränkungen im Alltag ergab sich aus den Erzählungen über gesundheitliche Einschränkungen im Alltag und wurde zur weiteren Auswahl von Fällen im Sinne des theoretischen Samplings während der Analyse von der Studienautorin zugeordnet. Die Datenanalyse folgte der dokumentarischen Methode nach Bohnsack [2]. Zunächst wurden die Audioaufnahmen auf Deutsch transkribiert. Anschließend wurde mithilfe der Analysesoftware MAXQDA 2020 (VERBI – Software. Consult. Sozialforschung. GmbH, Berlin, Deutschland) eine formulierende und reflektierende Interpretation durchgeführt.

## Ergebnisse

Alle Interviewten erzählten von meist mehreren Einschränkungen und Verlusten, die ihren Alltag grundlegend verändert haben, wie etwa Herzinfarkte, Schlaganfälle, Krebs, Verwitwung oder zitternde Hände. Die gesundheitlichen und sozialen Veränderungen werden dabei mit ihrem Alter erklärt und als fortschreitender Prozess erlebt.

„Wenn dir nichts fehlen würde, wüsstest du auch nicht, dass du alt bist. Das ist meine Einstellung. Mit dem Alter musst du damit rechnen, es kommt etwas.“ (IP15 Herbert, 408)

Die Interviewten berichten von verschiedenen Alltagssituationen, in denen Familienmitglieder (IP3 Brunhild), Bekannte (IP2 Elsbeth; IP1 Heidi) und Fremde – wie etwa die Kellnerin im Restaurant oder Ärzt:innen (IP12, Hans) – die Veränderungen als normal für ein gewisses Alter bezeichnen.

„Was willst du denn, Oma? Ja? Das war einmal, Oma! Was willst du machen? Ja, ja, wie soll ich sagen. Es ist traurig alles, aber es ist, äh, es ist der normale Verlauf, denk ich, nicht? Ich habe meine Mama so lange am Ende draußen gehabt im Altersheim. Ich bin dreieinhalb Jahre fast jeden Tag hinaus, hab sie besucht, jetzt mag ich nicht hinaus gehen. Das hat mir so gereicht. Wenn du das siehst, wie arm die Leute sind.“ (IP7 Alma, 94)

„Ich fühle mich eigentlich da drinnen jünger, als ich bin. Das hat mir der Therapeut einmal gesagt: ‚Weißt du, Brunhild, dein Herz und dein Hirn sind jung, aber das Gestell wird alt.‘ Es stimmt ja, oder? Ja. Das kannst du nicht ändern.“ (IP3 Brunhild, 512)

Die Interviewten sind tagtäglich mit der Fremdzuordnung zu der Gruppe des vierten Alters konfrontiert und müssen daher die negativ-behafteten Vorstellungen von dieser Gruppe in ihrem Selbstbild verhandeln. Um eine Identifikation mit dem sozial abgewerteten Bild eines abhängigen, älteren Menschen zu verhindern, wenden die Interviewten verschiedene Strategien zu Abgrenzung und Aufwertung ihrer eigenen Position an. Die nachfolgende Analyse verdeutlicht dabei, dass das vierte Alter auch im Alltag der Interviewten als ein soziales Imaginär im Sinne von Higgs und Gilleard [8] fungiert, indem die Interviewten auf Distanz zu den kulturellen Vorstellungen des vierten Alters gehen und das vierte Alter anderen Menschen – insbesondere Pflegeheimbewohner:innen – zuschreiben. Dies geschieht nicht nur durch eine Abgrenzung von anderen Menschen, sondern auch, indem die Erwartungen an sich selbst und das eigene Selbstbild angepasst werden und die Kommunikation gegenüber anderen selektiert wird. Im Folgenden werden die vier zentralen Strategien näher erläutert, nämlich (1) der Vergleich mit anderen älteren Menschen, (2) die Betonung der eigenen Leistungsfähigkeit, (3) die Neudefinition der eigenen Autonomie und (4) die selektive Kommunikation.

### Abgrenzung von anderen, älteren Menschen

Eine zentrale Strategie, um sich nicht als Teil der Gruppe des vierten Alters zu verstehen, stellt die Abgrenzung von Anderen dar, die die Interviewten dieser Gruppe zuordnen. Das können Verwandte oder Bekannte sein, die früh gestorben oder stark pflegebedürftig sind, aber auch generell Pflegeheimbewohner:innen.

„Unsere ehemalige Nachbarin, die hat nie was getan, gell? Die ist immer im Sommer im Liegestuhl draußen gelegen oder hinten draußen gelegen, die sind nur dagesessen. Wie wir laufen gegangen sind, sind sie auch laufen gegangen, aber erst dann. Wenn wir Radfahren gegangen sind, sind sie auch Radfahren gegangen. Sie können nichts selbstständig tun. Und was ist? (…) Hat man sie ins Spital gebracht, was hat sie? Herzbeschwerden, aber wie! Aber hätte sie etwas getan, das wäre auch besser für sie gewesen.“ (IP13 Maria, 539)

„Ja, jetzt bin ich 80. Ich bin zufrieden, so wie es ist, weil ich schau manchmal auf andere 80-Jährige, und das ist Wahnsinn, wie die dastehen, und da bin ich so zufrieden und dankbar. Und da sage ich auch danke für jeden Tag! Danke, dass ich das halt wieder geschafft habe.“ (IP2 Elsbeth, 197)

„Abends schlafen gehen und dann einschlafen, das wäre das Schönste, gell? Nur nicht lang leiden, wenn du so schaust in den Altersheimen. Wir haben a großes Altersheim in der Stadt, aber das ist, da wäre es schon besser, wenn man einschlafen könnte, gell?“ (IP16 Selina, 390)

Die Interviewten gehen davon aus, dass man im Alter mit Krankheiten rechnen muss, jedoch können diese in Grenzen gehalten werden, wenn man sich aktiv damit auseinandersetzt. Gesundheit und Krankheit im Alter liegen also im eigenen Verantwortungsbereich, und daher sehen die Interviewten die Schuld für eine Verschlechterung der Gesundheit bei der Person selbst. Herbert (IP15) erzählt, wie er kurzfristig „gleichgültig“ gegenüber seinem alternden Körper war.

„Irgendwie Gleichgültigkeit, dass in dem Alter, wenn man so arbeitet, man mit dem Herz Unterstützung machen muss. Da war ich gleichgültig. (…) Und plötzlich bin ich die Stiege ohne Stehenbleiben und Atmen nicht mehr durchgekommen. ‚Ja, was ist jetzt los?‘ Bin ich zum Doktor [Name] (…) und habe es ihm gesagt, hat der gelacht: ‚Eine Herzschwäche hast du.‘ (…) Das kommt halt mit dem Herz, und da musst du mit Crataegutt [Herz-Kreislauf-Medikament] gut dazuschauen. Das habe ich gemacht, und jetzt lauf ich die Stiegen wieder durch.“ (IP15 Herbert, 401)

Indem die Interviewten eine symbolische Grenze zwischen sich selbst und „den Anderen“ ziehen, können sie sich einer positiv bewerteten Gruppe zuordnen. Die soziale Kategorie des vierten Alters wird also Anderen zugeschrieben. Die Zuschreibung von Verantwortung oder Schuld an die Anderen verstärkt diese Differenzierung zusätzlich und stabilisiert die Wahrnehmung einer eigenen Überlegenheit.

### Betonung von Leistungsfähigkeit

Eine Identifikation mit den negativen Vorstellungen des vierten Alters wird jedoch nicht nur über eine Abgrenzung zu Anderen verhindert, sondern auch durch die Betonung der eigenen Leistungsfähigkeit. Für die Interviewten ist es von großer Bedeutung für ihr Selbstbild, ihre „Arbeit leisten“ zu können (IP7 Alma, 86). Dazu gehören vor allem das Kochen und Putzen (IP2 Elsbeth, IP8 Theresa, IP12 Hans), aber auch das Autofahren (IP11 Gerald, IP14 Franz) und das Nachgehen von Hobbies, wie etwa Spazierengehen (IP9 Stefanie) oder Handwerken (IP11 Gerald). Ein wichtiger Bestandteil, um eine positive soziale Identität für sich herzustellen, ist es, einen eigenen Beitrag zu leisten, der idealerweise mit sozialer Anerkennung einhergeht. So betont Selina, die gemeinsam mit ihrem Sohn und seiner Familie auf einem Bauernhof lebt, dass das Käsemachen ihr alleiniges „Geschäft“ ist, wodurch sie sich immer noch als Teil des „Wir“ versteht.

„B: Wir haben Wild, wir haben Schwein, wir haben Rind, wir haben alles! Wie gesagt, wir sind Selbstversorger. Wir haben Milch, wir haben Butter, wir machen Schafskäse, aber aus der Kuhmilch, was alle gern essen, wir machen es eh für uns, wir verkaufen es nicht, gell?

I: Tun Sie da auch noch mithelfen? Beim Käsemachen und so?

B: Das mache ich allein, den Käse! Das ist mein Geschäft! (…) Früher habe ich in der Landwirtschaft da noch gearbeitet und da und da. Das mache ich heute nicht mehr. Das macht heute die Jugend.“ (IP16 Selina, 379)

### Neudefinition von Autonomie

Ähnlich wie in den Arbeiten von Lloyd et al. [12], konnte auch hier eine „relationales Autonomieverständnis“ festgestellt werden. Da die Interviewten diverse körperliche Einschränkungen aufweisen, ist für eine Abgrenzung vom vierten Alter eine Neudefinition von Autonomie und Selbstständigkeit notwendig. Um ein positives Selbstbild herzustellen, wird Autonomie in Relation zu anderen Menschen und Artefakten verstanden, wie etwa Taxifahrern, Angehörige, Notrufarmbänder oder Gehstöcken.

„Und 2‑, 3‑mal, da geht die älteste Tochter mit mir zu der jüngsten nach [Ort], das mach ich schon. Allein täte ich es nicht mehr, glaube ich. Ich täte den Zug nicht finden. Das ist nicht mehr einfach. Aber wenn wir nach [Ort] gehen, da hat die jüngste Tochter schon alles organisiert, und den Zug hat die älteste Tochter organisiert. Der Papa muss bloß noch die Geldtasche mitnehmen. Weil wenn ich in [Ort] bin, da brauchen meine Töchter kein Geld, das macht der Papa! Das lass ich mir nicht nehmen. Wenn sie auch 50 sind, aber es sind immer noch meine Kinder. Das lass ich mir nicht nehmen.“ (IP12 Hans, 294)

Da körperliche Einschränkungen objektiv eine Nähe zur negativ konnotierten Kategorie des „vierten Alters“ herstellen könnten, wird die Bedeutung von Autonomie verschoben: Nicht vollständige Unabhängigkeit, sondern die Fähigkeit, Unterstützung aktiv zu organisieren und zu steuern, wird zum zentralen Kriterium der Selbstdefinition. Durch diese Neubewertung können sich die Interviewten weiterhin der Gruppe der „autonomen“ Älteren zuordnen und zugleich Distanz zu jenen herstellen, die sie als vollständig abhängig beschreiben.

### Selektive Kommunikation

Schließlich ist eine weitere Strategie, nicht alles zu kommunizieren. Mehrere Interviewte erzählen, wie sie insbesondere ihren Kindern, aber auch ihren Ärzt:innen verheimlichen, wie es ihnen zeitweise geht. Theresa (IP8) nimmt lieber weiter Schmerzmittel, um ihren Haushalt machen zu können und keinen Grund für einen Pflegeheimantrag zu liefern, obwohl ihre Ärzt:innen ihr sowohl von den Schmerztabletten als auch vom Putzen abgeraten haben. Hilde (IP10) empfindet ihre Kinder als bevormundend und würde ihnen daher nie von schlechteren Phasen erzählen. Auch das selektive Kommunizieren dient dazu, nach außen das Bild des „nichtpflegebedürftigen“ Älteren zu bewahren.

„Und ich habe natürlich Phasen, wo es mir ganz mies geht. Ah, werde ich meinen Kindern nie sagen, weil, das ist immer eine Katastrophe dann, und helfen kann mir eh keiner, aber dann bleib ich halt liegen, nimm doppelte Pulver und meinen Spray, und was ich halt da alles hab, und gib eine Ruh.“ (IP10 Hilde, 646)

## Diskussion

Die Ergebnisse zeigen, dass die Interviewten vielfältige Strategien entwickeln, um sich von der Fremdzuschreibung des negativ-konnotierten vierten Alters zu distanzieren und für sich selbst eine positive soziale Identität aufrechtzuerhalten. Einerseits können dabei die Annahmen von Higgs und Gilleard [8–10] über das vierte Alter durchaus bestätigt werden. Denn auch bei den Interviewten lässt sich das vierte Alter als soziales Imaginär ausmachen, dass sie für sich selbst ablehnen und anderen Personen von außen zuschreiben. Die eigene Ablehnung der Fremdzuschreibung erfolgt dabei nicht, indem sie sich selbst als gesund und aktiv darstellen oder erleben, sondern indem sie eine weitere Differenzierung quasi innerhalb des vierten Alters vornehmen. Denn die vorliegende Analyse legt nahe, dass die Interviewten durchaus ihre Einschränkungen und Verluste im Kontext von defizitären Vorstellungen über das Altern erleben und verhandeln, jedoch Autonomie und Selbstständigkeit als entscheidenden Unterschied heranziehen. Während sie sich selbst der eingeschränkten, aber autonomen Gruppe zuordnen, werden andere ältere Menschen der eingeschränkten und nichtautonomen Gruppe zugeordnet. Ähnliche Ergebnisse verdeutlicht die Studie von Pirhonen et al. [16], wobei Funktionalität und Autonomie die entscheidenden Vergleichskriterien mit anderen darstellen.

Denn obwohl das vierte Alter als soziales Imaginär verstanden werden kann, realisiert es sich erst durch die Fremdzuschreibung zu konkreten Personen. So zeigt sich, dass die eigene Fremdzuschreibung des vierten Alters abgelehnt wird, indem die defizitären Vorstellung anderen Personen zugeschrieben wird, allen voran Pflegeheimbewohner:innen. Das Pflegeheim nimmt dabei eine besondere Rolle ein. Gilleard und Higgs [4] beschrieben das Pflegeheim als eine Materialisierung der Vorstellungen über das vierte Alter, wobei die hier interviewten Personen das Pflegeheim als materiellen Marker verwenden, um sich von der Gruppe der Eingeschränkten und Nichtautonomen abzugrenzen.

Im Sinne der Theorie der sozialen Identität von Tajfel [22] erfolgt die eigene Zuordnung zur „autonomen“ Gruppe nicht nur durch Abgrenzungsstrategien von anderen Personen, sondern auch durch Aufwertungsstrategien der eigenen Position. So erfolgt die eigene Zuordnung zur „autonomen“ Gruppe, indem die Interviewten ihre eigene Autonomie trotz ihrer Einschränkungen und Verluste betonen und ihre Leistungsfähigkeit hervorheben. Ein neues Verständnis von Autonomie ist dabei zentral für eine Aufwertung der eigenen Position. Dies entspricht im Wesentlichen den Ergebnissen von Lloyd et al. [12]: Die Interviewten erleben sich nicht trotz, sondern teilweise gerade durch die Nutzung von Unterstützung als autonom, solange sie diese Unterstützung selbst organisieren und kontrollieren können.

Neben der Autonomie spielt zudem das Erleben von Leistungsfähigkeit eine zentrale Rolle für das Selbstverständnis der Interviewten. Gesellschaftliche Diskurse über Selbstständigkeit und Leistungsfähigkeit können stigmatisierend wirken, wenn man diesen Diskursen nicht mehr entsprechen kann [17, 21]. So zeigen Studien, dass hilfsbedürftige ältere Menschen sich deswegen schuldig und minderwertig fühlten [17]. Vorstellungen vom vierten Alter und der Umgang mit der Fremdzuschreibung scheinen also kulturell geprägt zu sein. Weitere Forschungen zum vierten Alter könnten kulturübergreifend ansetzen und insbesondere Kulturen heranziehen, in denen der Leistungsgedanke nicht so tief verwurzelt ist.

Schließlich selektieren die Interviewten sehr genau, welche Informationen sie über ihren Gesundheitszustand teilen. Dies lässt sich im Sinne von Goffmans Stigma-Theorie [5] interpretieren, bei der Individuen strategisch entscheiden, was sie erzählen, um eine stigmatisierende Information über sich zu kontrollieren und damit ihre soziale Identität zu schützen. Gerade im Kontext medizinischer und pflegerischer Interaktionen mit älteren Menschen ist diese Dynamik bedeutsam, da Fragen von Autonomie und Abhängigkeit identitätsrelevante Bedeutungen annehmen können. Werden diese Aspekte in der Interaktion berücksichtigt, kann dies dazu beitragen, Vertrauen zu stärken und die selektive Weitergabe gesundheitsbezogener Informationen zu reduzieren.

## Fazit für die Praxis

- Einschränkungen im Alter werden als Bedrohung der sozialen Identität wahrgenommen, und daher kommt der Selbstständigkeit und Autonomie große Bedeutung zu, sodass vielfach Informationen nicht kommuniziert werden, um die Identität zu schützen.
- Für die medizinische und pflegerische Praxis bedeutet dies, dass Empfehlungen, die zentrale Tätigkeiten vollständig einschränken, aus Sicht der Betroffenen als Bedrohung ihrer Identität erlebt werden.
- Autonomiebedürfnisse ernst zu nehmen und eine vertrauensvolle Kommunikation aufzubauen, könnte hier einen wichtigen Ansatz darstellen.

## Data Availability

Die zugrunde liegenden Daten sind aus Datenschutzgründen nicht öffentlich zugänglich. Auf Anfrage kann jedoch eine Kooperation entstehen.
